# QTM: Computational package using MPI protocol for Quantum Trajectories Method

**DOI:** 10.1371/journal.pone.0208263

**Published:** 2018-12-10

**Authors:** Marek Sawerwain, Joanna Wiśniewska

**Affiliations:** 1 Institute of Control & Computation Engineering, Faculty of Computer, Electrical and Control Engineering, University of Zielona Góra, Zielona Góra, Lubusz Voivodeship, Poland; 2 Institute of Information Systems, Faculty of Cybernetics, Military University of Technology, Warsaw, Masovian Voivodeship, Poland; Vilvius University, LITHUANIA

## Abstract

The Quantum Trajectories Method (QTM) is one of the frequently used methods for studying open quantum systems. The main idea of this method is the evolution of wave functions which describe the system (as functions of time). Then, so-called quantum jumps are applied at a randomly selected point in time. The obtained system state is called as a trajectory. After averaging many single trajectories, we obtain the approximation of the behavior of a quantum system. This fact also allows us to use parallel computation methods. In the article, we discuss the QTM package which is supported by the MPI technology. Using MPI allowed utilizing the parallel computing for calculating the trajectories and averaging them—as the effect of these actions, the time taken by calculations is shorter. In spite of using the C++ programming language, the presented solution is easy to utilize and does not need any advanced programming techniques. At the same time, it offers a higher performance than other packages realizing the QTM. It is especially important in the case of harder computational tasks, and the use of MPI allows improving the performance of particular problems which can be solved in the field of open quantum systems.

## 1 Introduction

The Quantum Trajectories Method (QTM) is an important method actively applied for investigation in the field of open quantum systems [1], [2], [3], [4]. It was implemented as packages in a few programming languages and tools. The first implementation was a package written by Sze M. Tan for the Matlab environment [5]. There is also a package for the C++ language [6], [7]. The latest implementations are QuTIP [8], [9] for the Python Ecosystem and QuantumOptics.jl [10] prepared for the Julia language.

The aforementioned solutions, especially QuTIP and QuantumOptics.jl, allow utilizing parallel computing inside the environments of Python and Julia. However, the packages are not intended for High-Performance Computing (HPC) [11], [12] where Message Process Interface, termed as MPI [13], plays a significant role. Using MPI allows us to re-implement the QTM for HPC systems, regardless of their scale (the QTM is scale-free because each trajectory may be calculated separately). The scale-free character of the QTM will allow utilizing more computing power, and that will result in shorter time of calculations, which is especially important for cases in which 20,000 and more trajectories are generated.

Previously, we prepared the implementations of the QTM for CPUs and GPUs [14], [15]. The results presented in this work relate to a brand new implementation of the QTM for the MPI protocol (the actual source code of the QTM can be found at [16]). The version 1.0a of the QTM package is also available at [17].

As far as the QTM is concerned, a proper selection method for solving systems of Ordinary Differential Equations (ODEs) must be considered. This is a basic action taken while numerical computations are realized. More precisely, a very important issue is an Initial Value Problem (IVP). Due to that, the selection of an appropriate method for solving IVP (in general ODEs) is crucial. Especially when the system of equations is difficult to solve, e.g. so-called systems of stiff ODEs. The stiff ODEs constitute a significant type of ODEs and the correct solving of these equations is pivotal for numerical computations in many cases—especially for the QTM where calculating many trajectories requires solving many ODEs.

A few groups of methods [18], [19] used in the context of stiff ODEs may be recalled, and these are the Backward Differentiation Formula (BDF) methods. Another approach, which is commonly used in numerical computations for solving ODEs, is Livermore Solver for ODE (LSODE) method [20]. In this work, we reuse the LSODE variant called ZVODE (LSODE for complex numbers) for the implementation of the QTM in the MPI environment.

The ZVODE package is a stable solution offering high accuracy, but this is not a reentrant solution which may be directly utilized in a parallel environment. However, this problem can be solved by utilizing MPI where the processes are separate programs communicating with one another using message passing. Therefore, one MPI process calls only one instance of the ZVODE method.

The ZVODE package was implemented in the Fortran language. The QTM package, implemented in the C++ language, uses the ZVODE package efficiently. However, we made some effort to assure that functions offered by the QTM package are easily accessible as it is in the packages QuTIP and QuantumOptics.jl. Naturally, programs (delivered as examples or written by the user) utilizing the QTM have to be compiled. This should not be a problem because of used makefile mechanism for simplifying this process.

The paper is organized in the following way: in Section 2, we shortly present selected mathematical features of the QTM. The BDF numerical methods for solving IVP, used in the presented package are discussed in subsection 2.1. Whereas in 2.2 the algorithm for the QTM is presented. There are also some remarks pointing out where the parallel processing techniques and the MPI technology are utilized.

Section 3 contains selected remarks referring to the implementation of the QTM package. In this section, the most important data types implemented in package are presented. We also describe how the ZVODE package is used to solve ODEs since solving ODEs poses a significant problem in the QTM.

We analyze the efficiency of our implementation in a comparison with other recently developed packages in section 4. The most important issue presented in this section is the scalability of the QTM.

A summary of achieved results is discussed in Section 5. There are also presented further aims which are planned to be realized as the next steps in the evolution of the demonstrated implementation of the QTM. The article is ended with acknowledgments and bibliography.

## 2 Quantum Trajectories Method

Performing the QTM requires solving ODEs. The presented solution, as mentioned above, uses the ZVODE package which allows utilizing some numerical methods indispensable for the proper functioning of the QTM. The fundamental information concerning the ZVODE package is presented in subsection 2.1, while subsection 2.2 contains the description of the QTM, including a method of calculating a single trajectory [14], [15] with the use of MPI technology. The properties of the QTM allow us to easily distribute the computations—this feature helps to accelerate the calculations and makes the process scalable.

Another important element of the solution is a pseudorandom number generator. In subsection 2.3, we describe the generator which we chose to utilize in the implementation of the QTM package.

### 2.1 Methods for solving ODEs

The ZVODE package was built on a basis of the VODE package [21]. The VODE package was based on the LSODE package. The numerical methods, used for the LSODE (Livermore Solver for Ordinary Differential Equations) package implementation, are the computational routines based on the group of Linear Multistep Methods (LMMs). In general, LSODE uses the Adams methods (so-called predictor-corrector methods) for the non-stiff ODEs solving. In case of stiff ODEs, the Backward Differentiation Formula (BDF) methods are used. In this section, the most important mathematical aspects of the aforementioned methods are briefly presented—the detailed report concerning standard (i.e. sequential) implementation of LSODE may be found in [22].

The choice of the ZVODE package is determined by the fact that the QTM needs using complex numbers and the ZDOVE package allows performing calculations on complex numbers of single and double precision.

For the Initial Value Problem (IVP) in general:

$$\begin{array}{c} \begin{array}{ccc} y^{\prime } & = & f(x,y),\\ y(x_{0}) & = & y_{0}. \end{array} \end{array}$$ (1)

The LMMs, approximating the problem’s solution, may be described as:

$$\begin{array}{c} \sum_{i=0}^{k}\alpha_{\left(k-i\right)}y_{\left(n+1-i\right)}=h\sum_{i=0}^{k}\beta_{\left(k-i\right)}f\left(x_{\left(n+1-i\right)},y_{\left(n+1-i\right)}\right), \end{array}$$ (2)

where $\alpha_{i},\beta_{i}\in \mathbb{R}$ and *α*_*k*_ ≠ 0 and |*α*_0_| + |*β*_0_| > 0. The parameter *h* represents the step width for integration. Naturally, a value of *h* is selected during the solver’s work with the use of adaptive methods.

The LSODE routine uses two methods based on the LMMs: the Adams-Moulton method and the BDF method.

The implicit Adams-Moulton method, this means the values of *β*_*i*_ ≠ 0 (the detailed description of used symbols may be found in [23]), may be presented as:

$$\begin{array}{c} y_{n+1}=y_{n}+h\sum_{i=0}^{k}\gamma_{i}\nabla^{i}f_{n+1}, \end{array}$$ (3)

where

$$\gamma_{i}={\left(-1\right)}^{i}\int_{0}^{1}\left(\begin{array}{c} -s+1\\ i \end{array}\right)ds.$$ (4)

The ∇^*i*^ symbol stands for the backward differences. It should be pointed out that: ∇^*i*^ *f_n_* = *f_n_* and ∇^*i*+1^ *f_n_* = ∇^*i*^ *f_n_* − ∇^*i*^ *f*_*n*−1_. The value *k* expresses the number of steps, i.e. the values of *y*_*i*_ used in the specified method.

The BDF method is defined as:

$$\begin{array}{c} \sum_{i=1}^{k}\frac{1}{i}\nabla^{i}y_{n+1}=hf\left(x_{\left(n+1\right)},y_{\left(n+1\right)}\right). \end{array}$$ (5)

The above notation means that *β*_*k*_ ≠ 0 but *β*_*i*_ = 0 for *i* = 0, 1, 2, …, *k* − 1. The values of *α*_*i*_ are arbitrarily defined and may be found in many publications concerning the methods of solving ODEs.

In both cases, for the Adams-Moulton and the BDF method, the problem is to estimate the value of *f*_*n*+1_. It should be stressed that this value is needed to estimate itself. The Newton method [24] is used in this case to calculate the estimation. The Newton method is quite rapidly convergent, though for the system of equations it needs to calculate the Jacobian. This method for (ZV/LS)ODE may be expressed as:

$$\begin{array}{c} g\left(y_{n+1}^{\left(1\right)}\right)=-(I-\frac{\partial f}{\partial f})\left(y_{n+1}^{\left(2\right)}-y_{n+1}^{\left(1\right)}\right), \end{array}$$ (6)

where $y_{n+1}^{\left(2\right)}$ and $y_{n+1}^{\left(1\right)}$ stand for the next approximations of *y*_*n*+1_. The function *g*(⋅) represents the function approximating values of *y*_*n*+1_.

**Fig 1:**
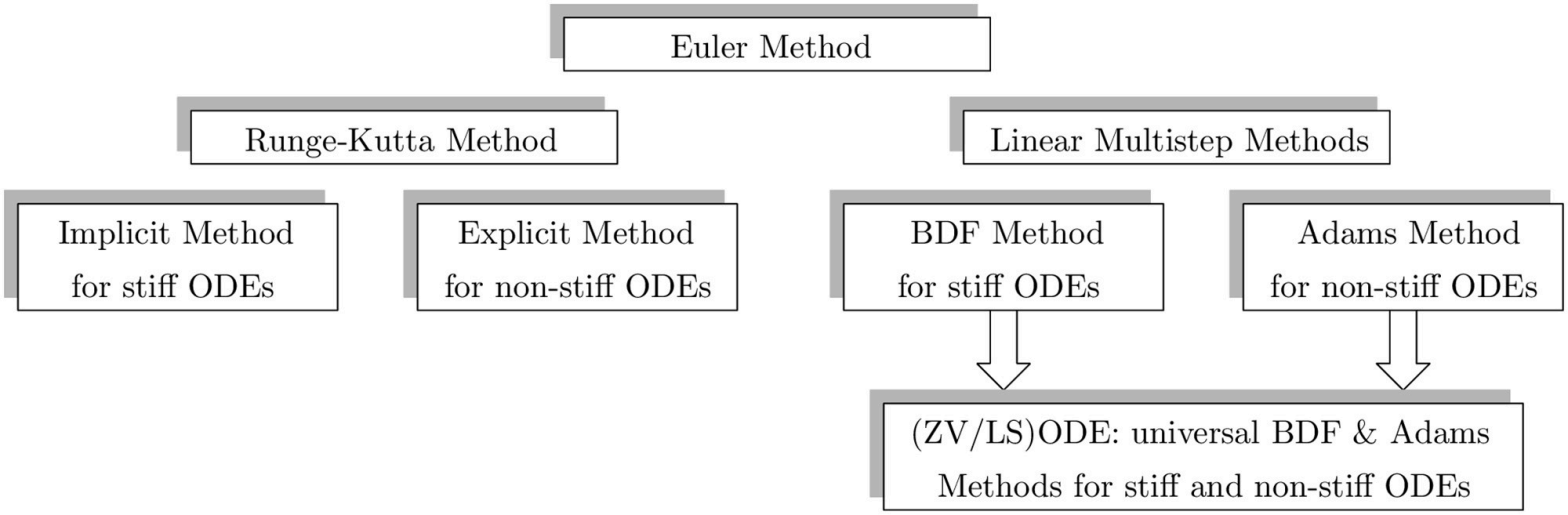
A general overview of the ODE solver and the methods used in the (ZV/LS)ODE implementation.

The BDF method is used for stiff ODE problems and the Adams-Moulton method for non-stiff ODE problems. Using these both approaches together makes the (ZV/LS)ODE a hybrid method—see Fig 1. The presented QTM implementation allows choosing the method, respectively to solving easier (non-stiff ODE) or more difficult (stiff ODE) QTM problems. Moreover, in ZVODE, for every method the method’s order can also be controlled automatically. For the Adams-Moulton method, the orders from the first to the twelfth are available, and for the BDF method the orders from the first to the sixth.

### 2.2 Quantum Trajectories Method

A description of quantum states’ dynamics may be presented for two fundamental situations. The first case is an evolution of a closed quantum system, whereas the second case concerns an evolution of an open quantum system. As an example of a closed quantum system, we may refer to a model of a quantum circuit. If we want to consider a quantum system where its dynamics is affected by the influence of an external environment, we deal with an open quantum system.

In this subsection, we do not aim to describe the mathematical models of dynamics in open and closed quantum systems—the details concerning this subject may be found in [4] and [25]. For clarity, it should be recalled that for closed systems, the evolution is a unitary operation, and it can be denoted as Schrödinger equation:

$$(C1):\;i\hbar \frac{\partial }{\partial t}\Psi =\hat{H}\Psi ,\;\;(C2):\;i\hbar \frac{d}{dt}|\psi \rangle =H|\psi \rangle ,$$ (7)

where (C1) is the form of a partial differential equation and (C2) is the form which is convenient to use in numerical simulations. In (C2) *H* is a Hamiltonian describing system’s dynamics, and |*ψ*〉 stands for the initial system’s state.

The von Neumann equation describes the quantum system’s evolution if an influence of an external environment has to be considered:

$$\begin{array}{c} {\dot{\rho }}_{\text{tot}}\left(t\right)=-\frac{i}{\hbar }\left[H_{\text{tot}},\rho_{\text{tot}}\left(t\right)\right],\;\;\;H_{tot}=H_{sys}+H_{env}+H_{int}, \end{array}$$ (8)

where *H*_*sys*_ denotes the dynamics of a closed/core system, *H*_*env*_ stands for the environment’s dynamics, and *H*_*int*_ describes the interaction’s dynamics between the external environment and the system. The environment’s influence can be removed from (8) by the partial trace operation. In such a case, we obtain an equation which describes the dynamics of the core system. Such a system can be expressed by the Lindblad master equation:

$$\begin{array}{c} \dot{\rho }\left(t\right)=-\frac{i}{\hbar }\left[H\left(t\right),\rho \left(t\right)\right]+\sum_{n}\frac{1}{2}[2C_{n}\rho \left(t\right)C_{n}^{+}-\rho \left(t\right)C_{n}^{+}C_{n}-C_{n}^{+}C_{n}\rho \left(t\right)], \end{array}$$ (9)

where *C*_*n*_ stands for a set of collapse operators. These operators represent the influence of an external environment which affects a simulated system. Naturally, applying a collapse operator causes irreversible modification of a quantum state. It should be emphasized that simulating the behavior of a quantum system needs exponentially growing memory capacity according to the system’s dimension.

The QTM is a method which facilitates reducing the memory requirements during the simulation. Of course, any simulation of a quantum system’s behavior needs calculating many single trajectories—the more, the better—because they will be averaged to one final trajectory, and a greater number of trajectories ensures improved accuracy. However, every trajectory may be simulated separately, and this fact provides a natural background to utilize a parallel approach while implementing the QTM.

If we would like to compare simulating the behavior of a quantum system with the use of Lindblad master equation and the QTM, we should consider the requirements of both methods on computational resources. The Lindblad master equation methods utilize the density matrix formalism and the QTM is based on a wave function of *n*-dimensional state’s vector (termed as a pure state). A number of this vector’s entries grows exponentially, but using sparse matrices facilitates efficient simulation of a quantum system’s behavior. It should be stressed that a simulation based on the wave function concerns only one state of a quantum system what seems to be a disadvantage of this solution because for the Lindblad master equation methods, the density matrix describes many different states of the same system. Unfortunately, using density matrices in most cases is not possible because of memory requirements—the size of the density matrix grows exponentially when the dimension of a simulated system increases. While the QTM enables monitoring the influence of an external environment on a quantum state by modifying the state’s vector with the use of a collapse operator.

For the QTM, an evolution of a quantum system is described by so-called effective Hamiltonian *H*_eff_, defined with the use of a set of collapse operators *C*_*n*_, and a system’s Hamiltonian *H*_sys_:

$$\begin{array}{c} H_{\text{eff}}=H_{\text{sys}}-\frac{i\hbar }{2}\sum_{n}C_{n}^{+}C_{n}. \end{array}$$ (10)

The set of operators *C*_*n*_ determines the probability of a quantum jump occurrence. This phenomenon is caused by a single collapse operator acting on a current quantum state of the system. A probability of a quantum jump occurrence is:

$$\begin{array}{c} \delta p=\delta t\sum_{n}\left\langle \psi \left(t\right)\right|C_{n}^{†}C_{n}\left|\psi \left(t\right)\right\rangle . \end{array}$$ (11)

If a quantum jump takes place, the system’s state at the moment of time (*t* + *δt*)—that is just after the collapse operation—can be expressed as:

$$|\psi (t+\delta t)\rangle =\frac{C_{n}|\psi (t)\rangle }{\sqrt{\langle \psi (t)|C_{n}^{†}C_{n}|\psi (t)\rangle }}.$$ (12)

Furthermore, if many collapse operators may be applied in the considered model, the probability of using *i*-th operator is:

$$P_{i}(t)=\frac{\langle \psi (t)|C_{i}^{†}C_{i}|\psi (t)\rangle }{\delta p}.$$ (13)

Of course, simulating the phenomenon of a system’s collapse needs a random numbers generator to ensure its probabilistic character (this issue is discussed in the following subsection).

Let us emphasize that all the considered calculations correspond to operations implemented on matrices and vectors within the QTM package. The needed matrices are usually band matrices. Namely, it is convenient, in terms of the memory consumption and the speed of calculation, to use sparse matrices. In consequence, the compressed sparse row (CSR) format may be utilized—it also gives an additional speed-up, especially when many matrix-vector multiplications have to be realized.

By summarizing the above remarks, we can formulate an algorithm which presents how a single quantum trajectory is calculated:

**Algorithm 1** *Computation of a single trajectory*

*The algorithm computing a single trajectory, according to the QTM, may be described as four computational steps*:

*(A) a value* *0* ≤ *r* < 1 *is computed by a pseudorandom number generator (r denotes the probability of a quantum jump occurrence);*
*(B) to get the state’s vector at the moment t the Schrödinger equation is integrated with the Hamiltonian* *H*_eff_ *(providing that the state’s vector norm has to be equal or greater to r*: 〈*ψ*(*t*)|*ψ*(*t*)〉 ≥ *r*);
*(C) if a quantum jump is realized, then the system’s state projection at the moment t, to one of the states given by* Eq (12), *is calculated. The operator C*_*n*_ *is selected to meet the following relation for the adequate n*: $\sum_{i=1}^{n}P_{i}(t)\geq r$ *and P*_*i*_(*t*) *is given by* Eq (13);
*(D) the state obtained by the projection of wave-function in the previous step is a new initial value corresponding to the moment of time t*; *next, the new value of r is randomly selected, and the procedure repeats the process of the quantum trajectory generation, starting from the step (B)—more precisely: the simulation is performed again, but starts from the previously given value of t*.

The presented approach is based on [8], [9], [14], [15], [26], [27].

### 2.3 Pseudorandom number generator

The QTM package described in this work utilizes a pseudorandom number generator from the Generalized Feedback Shift Register (GFSR) class. More precisely, we chose the LFSR113 method which is defined by using recurrence over the field *F*_2_ consisting of elements 0, 1:

$$\begin{array}{c} x_{n}=\left(a_{1}x_{n-1}+a_{2}x_{n-2}+\ldots +a_{k}x_{n-k}\right)\;\text{mod}\;2, \end{array}$$ (14)

where $a_{j_{1}}$ are generator’s parameters (*j*_1_ = 1, …, *k*) and $x_{j_{2}}$ are generator’s seeds (*j*_2_ = *n* − 1, …, *n* − *k*). The generator’s period is *r* = 2^*k*^ − 1 if and only if the characteristic polynomial of recurrence:

$$\begin{array}{c} P\left(z\right)=z^{k}-a_{1}z^{k-1}-\ldots -a_{k}, \end{array}$$ (15)

is primitive.

The generated values, for *n* ≥ 0, may be expressed as:

$$\begin{array}{c} u_{n}=\sum_{i=1}^{L}x_{ns+i-1}2^{-i}, \end{array}$$ (16)

where *s* denotes the step size, and *L* stands for the number of bits in a generated word. If (*x*_0_, *x*_1_, …, *x*_*k*−1_) ≠ 0, and *s* is coprime to *r*, then we obtain a periodic sequence of *u*_*n*_ (with a period denoted as *r*).

Of course, the quality of a generator is determined by the sequence *x*_*n*_. The proper choice of seeds is described in [28] where it is also shown that four seeds (*k* = 4) are sufficient to generate high-quality pseudorandom numbers. The LFSR113 generator’s realization is very fast because of the bitwise operations usage—this feature also does not collide with ensuring a sufficient period for generated numbers: 2^113^. However, it should be emphasized that the selection of generator’s seeds is crucial—there are four initial values, and they have to be integers greater than 1, 7, 15 and 127, respectively.

## 3 General implementation remarks

In the QTM, we calculate many independent trajectories. Because there is no relations between trajectories, it is easy to implement them utilizing parallel computing. Naturally, parallel computation shortens the time of calculations in comparison to serial computation. The calculated trajectories must be averaged to obtain the final trajectory.

**Fig 2:**
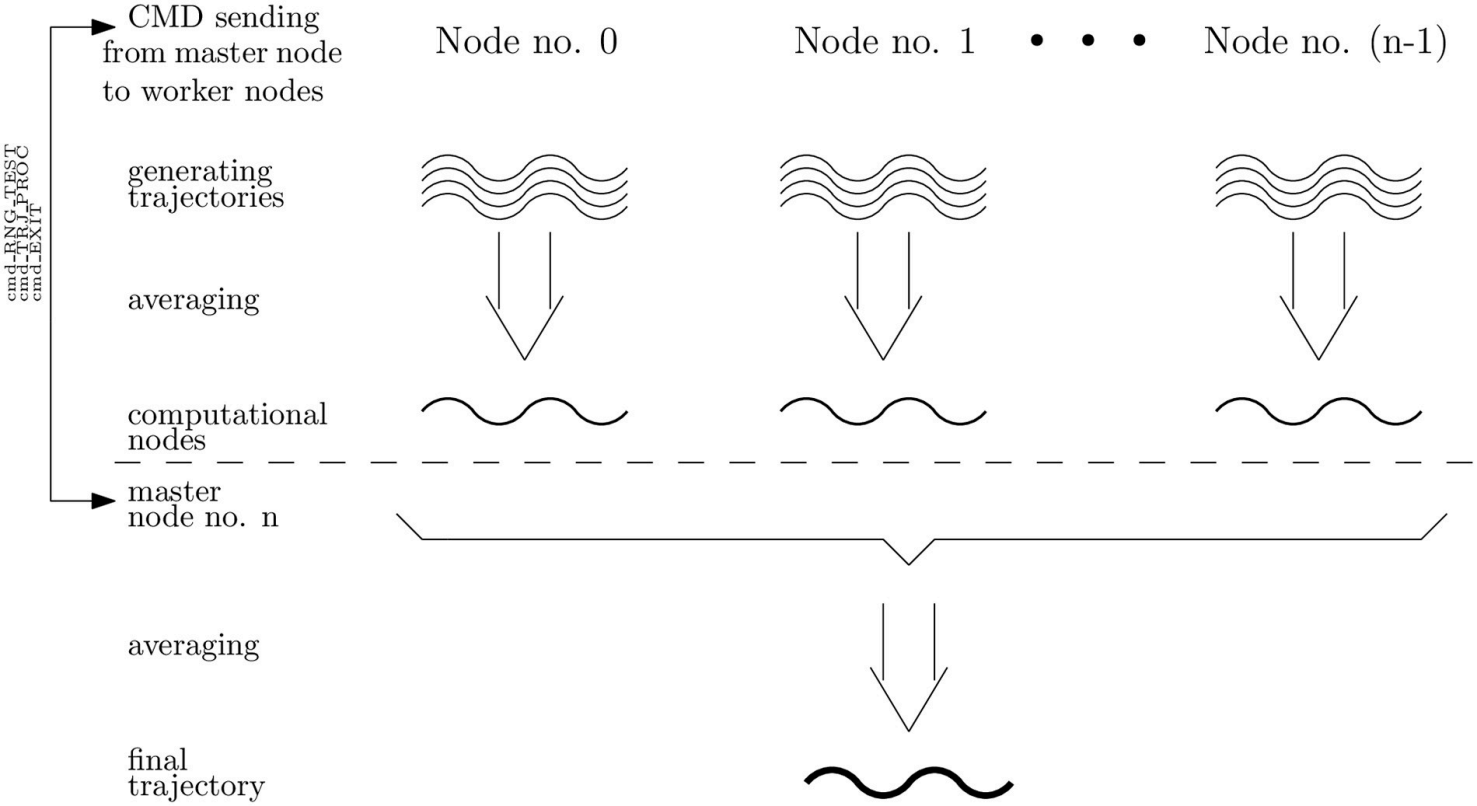
Computing and averaging trajectories with *n* + 1 computational nodes. We assume that *n* nodes calculate trajectories, and one node (denoted as node *n*) plays a master role—it coordinates the process of computations for the QTM. The figure presents also the general scheme of message passing during the realization of the QTM. The master node sends tasks, e.g. commands to calculate trajectories, and computing nodes response with results of their calculations.

Fig 2 depicts the idea of computing trajectories in many computational nodes. The exchange of necessary information between nodes is realized with use of MPI protocol. This protocol offers a scalable solution, what means that our QTM package works efficiently within a cluster of workstations, connected with the use of the Ethernet network, and also with one multi-core personal computer. In section 4, we show the acceleration of computations carried out with the use of the QTM package—the acceleration is noticeable regardless of whether we work with a cluster of workstations or with a one multiprocessor computer.

The process of calculating the final trajectory is presented in Fig 2. The diagram shows the flow of messages during the calculations. The messages are sent by the master node and other nodes respond to these messages. The main part of the calculations is preceded by some preliminary activities, e.g. initialization of pseudorandom numbers generators. Then, the body of calculations begins, and computational nodes calculate and average trajectories. The number of trajectories is influenced by the number of computational nodes and by the number of trajectories planned to be generated to simulate an analyzed problem. We assume an uniform load for every computational node. Each computational node calculates a number of trajectories and averages them. Finally, the averaged trajectories are sent to the master node where are averaged once again in order to obtain the final trajectory.

**Fig 3:**
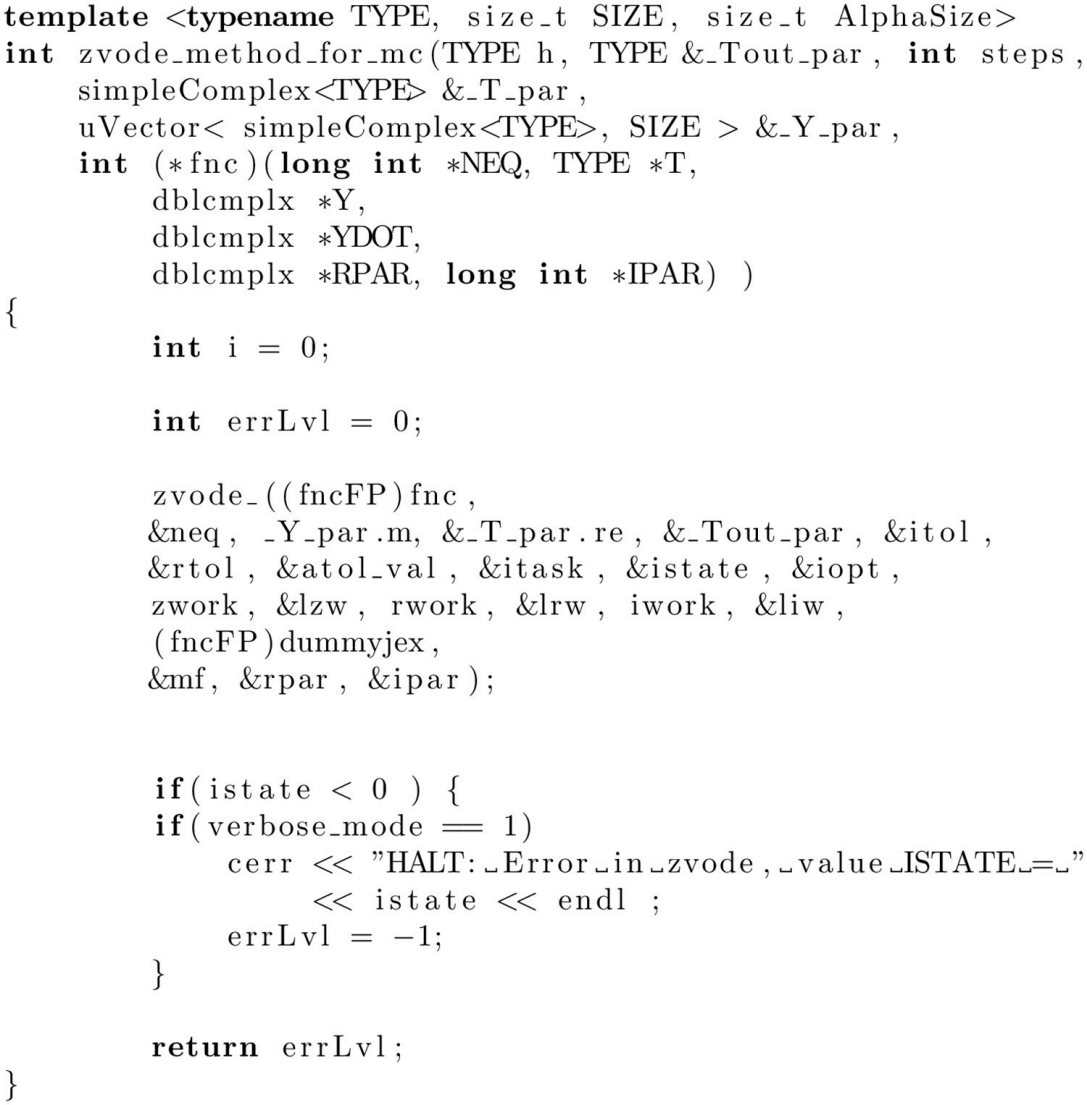
A function solving IVP (for a time-independent Hamiltonian) with the use of the zvode_ method from the ZVODE package. The underscore placed in the name of the method is a requirement imposed by the Fortran language.

Calculating one trajectory is directly connected with the ZVODE package. Despite the fact that this package was implemented in Fortran, the functions from the ZVODE library may be called in a code written in C++, just like other functions implemented in C++. To achieve that, we have to prepare an intermediary function based on a template. The exemplary function called zvode_method_for_mc is presented in Fig 3.

**Fig 4:**
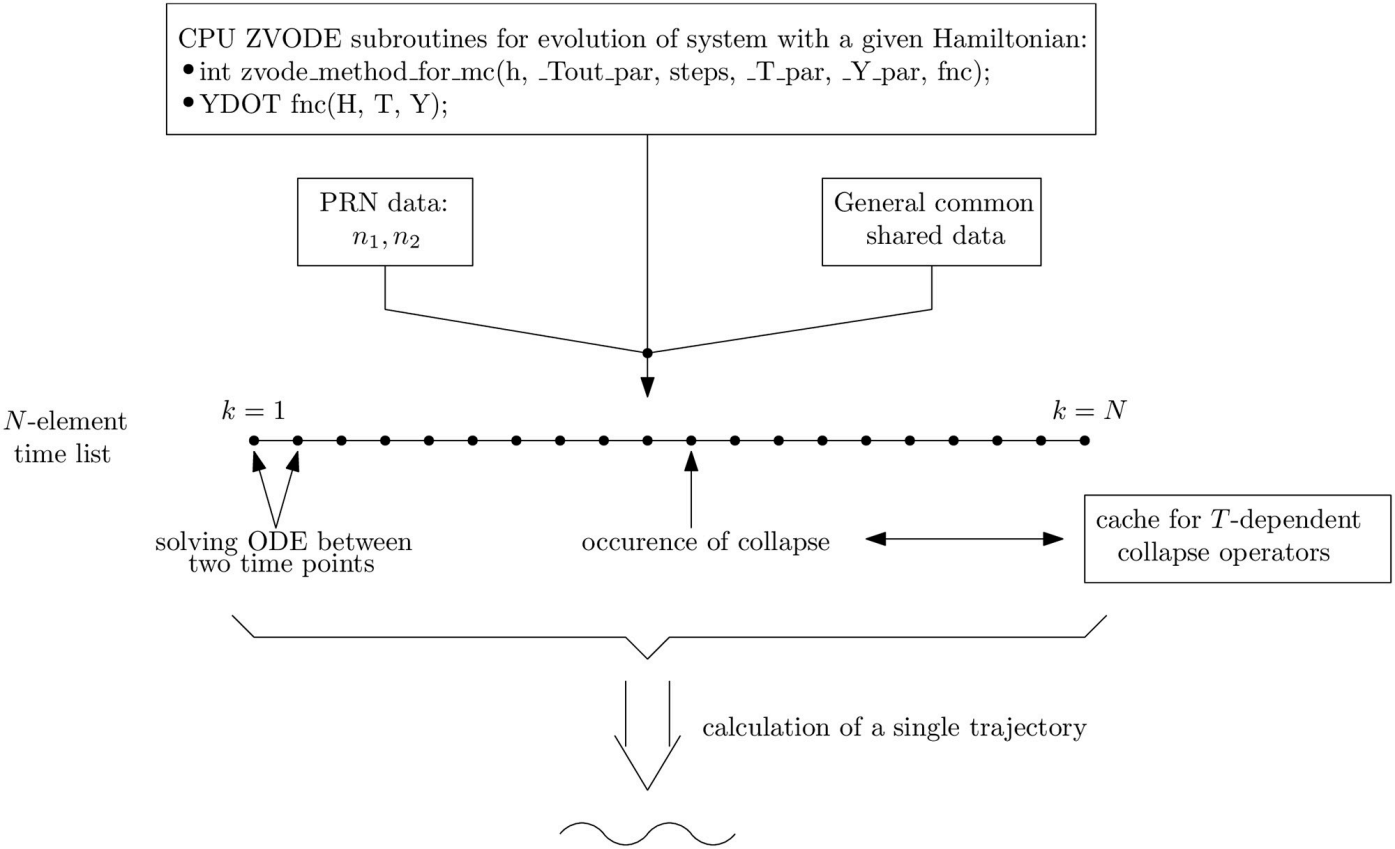
The general scheme presenting generation of a single quantum trajectory (PRN—Pseudo-random numbers; H—Hamiltonian data; zvode_method_for_mc—Subroutine to ODEs solving; _T_par—Time variable; _Y_par—Actual state of the system), fnc—Calculates the right side of ODEs.

The scheme of tasks realized during the calculation of a single trajectory, according to Algorithm 1, is shown in Fig 4. The algorithm is implemented in the C++ language, but it uses the zvode_method_for_mc method to solve a system of ODEs. The ZVODE package does not offer the reentrant property which allows many threads to utilize the same function (this property is realized by avoidance of using shared and global variables in function’s implementation). Therefore, one MPI process may call only one instance of any method from ZVODE. However, this feature does not pose a problem because we may run many MPI processes at the same time.

It is a very important assumption for our implementation that we use templates and static memory allocation (this applies to the code written by the user in order to, for example, describe some operators)—these techniques enable implementation of a code with an easier declaration of objects (the code is more similar to a code of a script language than to a C++ code with dynamic memory allocation). This makes using the package easier, but equally efficient. We even expect better efficiency because the static size of data structures is known during the compilation process, therefore, the compiler is able to optimize the numerical computation.

The QTM package offers three basic data types. The first one is simpleComplex < T >. It is dedicated to the operations on complex numbers, where T may be a float or a double. If we want to use simpleComplex < T > type together with the functions from the ZVODE library, then T is always double. The second type uVector < T > (intsize) is dedicated to vectors—the first parameter describes the type of vector elements, and a constructor parameter represents the number of entries. Similarly for matrices, a type uMatrix < T > (intsize) was introduced, where the first parameter describes the type of matrix elements, and the constructor parameter stands for the dimension (only square matrices are utilized in the QTM). Package also utilize the static and dynamic memory allocation. However, dynamic aspects of memory management were hidden in the implementation layer. Users of the QTM package are not obliged to create objects in dynamic approach.

An exemplary declaration of four matrices describing collapse operators is:

uMatrix< simpleComplex<**double**> > c_ops[4] = {{WV_LEAD_DIM}, {WV_LEAD_DIM}, {WV_LEAD_DIM}, {WV_LEAD_DIM}};

A value of WV_LEAD_DIM expresses, in this case, the dimension of operators and matrices.

In the QTM package, a uCSRMatrix < T > (size, rowptr, colind) type was also implemented. This type represents a column-row oriented sparse matrix. The sparse matrices are often utilized to describe processes taking place in open quantum systems. Using sparse matrices allows increasing the efficiency of computation and decreasing the amount of memory needed to hold, for example, collapse operators or expectation values. Furthermore, sparse matrices in CSR format offer a shorter time of multiplying these matrices by vectors.

The QTM package utilizes the MPI standard. However, after defining the initial structures describing a simulated problem, the user is not obliged to deal with details of the MPI protocol. The whole computational process is realized by a function mpi_main:

**template**<size_t N, size_t Ntrj, size_t _WV_LEAD_DIM,

    size_t _WV_LEAD_DIM_SQR, size_t _C_OPS_SIZE>

**int** mpi_main(**int** argc, **char** *argv[], **int** verbose_mode,

   **double** _from_time, **double** _to_time,

   **int** use_colappse_operator,

   **int** use_expecation_operator,

   extra_options opt);

In the above form of mpi_main function, the Hamiltonian is time-independent. If we would like the Hamiltonian to be time-dependent, in the call of mpi_main, we point an additional function which is used during the calculation of trajectories. The whole process of communication with the use of MPI is automatically realized within the mpi_main function. The parameters of the final trajectory may be directed to standard output or to a text file, what is determined by a value of the parameter opt. This parameter also serves to indicate the numerical method for solving ODEs. The ZVODE package offers two methods: ADAMS and BDF. A method is selected as below:

 opt.ode_method = METBDF;

or

 opt.ode_method = METADAMS;

The BDF method is dedicated to solving stiff ODEs but the ADAMS method may also be used in the QTM (sometimes it needs to calculate more trajectories or to increase the accuracy of the Adams’ ODEs solver).

## 4 Selected problems—Implementation and performance

During the realization of the QTM implementation, the essential task was to improve its performance in comparison to other existing QTM implementations. The usage of C++ programming language and especially the MPI technology facilitated us to obtain a stable solution with decent performance, thanks to the parallel processing of trajectories.

The performance of the presented solution was compared with two recently developed packages the QuTIP and the QuantumOptics.jl, which also fully supports the QTM (and the QuTIP also utilizes the ZVODE method). We have prepared two examples to examine the efficiency of the unitary Hamiltonian and the trilinear Hamiltonian. We also show computations referring to the Jaynes-Cummings model. The fourth example presents the results of the experiment also conducted in [29], and shows the accuracy of the QTM package.

### 4.1 Unitary Hamiltonian

The first example concerns a simulation of a system described by the following unitary Hamiltonian:

$$\begin{array}{c} H_{\text{SYS}}=\frac{2\pi }{10}\sigma_{x},\;\;\;\text{and}\;\;\;\sigma_{x}=[\begin{array}{cc} 0 & 1\\ 1 & 0 \end{array}], \end{array}$$ (17)

where *σ*_*x*_ represents Pauli operator *X* (also termed as the *NOT* operator). The initial state of the analyzed system is

$$\begin{array}{c} |\psi_{0}\rangle =|0\rangle =[\begin{array}{c} 1\\ 0 \end{array}]. \end{array}$$ (18)

The collapse operator and the expectation value operator, used in the simulation, are given below:

$$\begin{array}{c} C_{0}=\frac{5}{100}\sigma_{x},\;E_{0}=\sigma_{z},\;\;\;\text{and}\;\;\;\sigma_{z}=[\begin{array}{cc} 1 & 0\\ 0 & -1 \end{array}], \end{array}$$ (19)

where *σ*_*z*_ stands for the Pauli operator *Z*.

We ran the experiment on a PC equipped with the Intel i7-4950k 4.0 Ghz processor under the Ubuntu 16.04 LTS operating system. The utilized version of the QuTIP package is 4.2 and the QuantumOptics.jl ran on Julia 0.6.4. Although the dimensions of the above structures are quite small, the simulation of 1000 trajectories with the QuTIP package, when only one core is running, needs about ≈ 0.90 seconds. Utilizing e.g. two cores for the calculation does not reduce the time of process because the QuTIP needs time for coordinating two threads. Of course, with the greater number of trajectories, it is easy to observe that the time of simulation is shorter when more cores are active. If the QTM package is used, calculating 1000 trajectories with the use of e.g. of eight MPI computational nodes cores takes 2-3 seconds. This time is mainly consumed by starting the MPI processes. It should be mentioned that the QuantumOptics.jl package needs more time for calculation because of the JIT compiler usage. However, for a such small problems the time of calculation is almost the same, irrespective of the used package.

**Fig 5:**
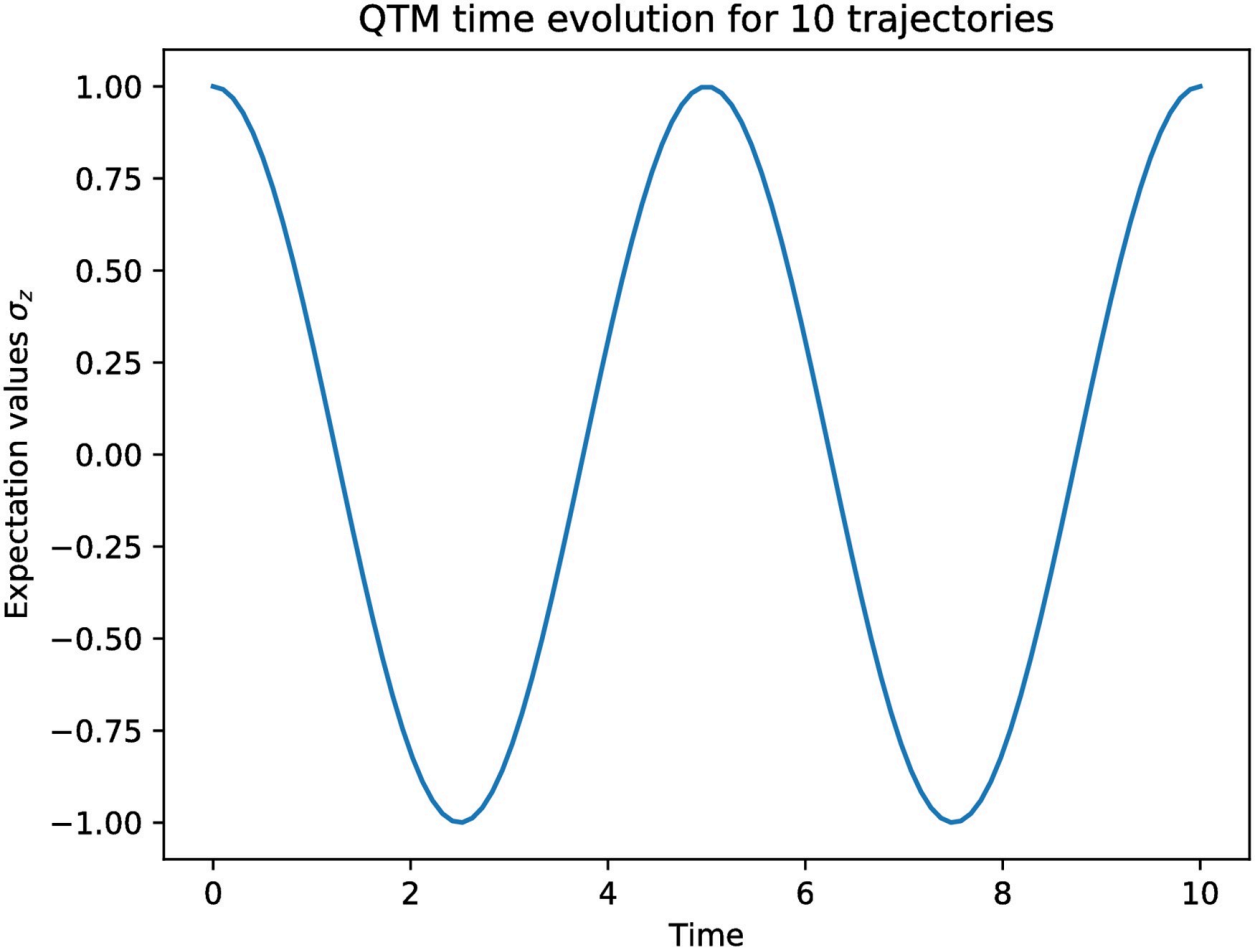
The figure showing changes of the unitary Hamiltonian’s expectation value in the time.

Fig 5 shows the evolution of the expectation value with the Pauli Z gate in time.

The most important pieces of the code are written in a direct form (we define an effective Hamiltonian form directly filling the matrix *H* with suitable entries) because the QTM package allows specifying data structures directly. Constants WD_LD and WD_LD_SQR respectively stand for the vector state dimension, and its square and they are equal to two and four; co represents the collapse operator, eo stands for the expectation operator value and H is the Hamiltonian:

**int** r = 0;

co[0] = msc(0.0, 0.0); co[1] = msc(0.05, 0.0);

co[2] = msc(0.05, 0.0); co[3] = msc(0.0, 0.0);

eo[0] = msc(1.0, 0.0); eo[1] = msc(0.0, 0.0);

eo[2] = msc(0.0, 0.0); eo[3] = msc(-1.0, 0.0);

alpha[0] = msc(1.0, 0.0);

alpha[1] = msc(0.0, 0.0);

H[0] = msc(-0.00125, 0.0);

H[1] = msc(0.0, -0.62831853);

H[2] = msc(0.0, -0.62831853);

H[3] = msc(-0.00125, 0.0);

c_ops[0].rows = 2; c_ops[0].cols = 2;

c_ops[0].m = co;

opt.type_output = OUTPUT_FILE;

opt.only_final_trj = 1;

opt.ode_method = METADAMS;

opt.tolerance = 1e-7;

opt.file_name = strdup(“output-data.txt”);

opt.fnc = &myfex_fnc_f1;

r = mpi_main<N, Ntrj, WV_LD, WV_LD_SQR, 1>(argc, argv, 1, 0, 10, 1, 1, opt);

A significant part of the source code is a function calculating the right side of the ODEs. In the case of the example for a unitary Hamiltonian, we may utilize a direct approach that is multiplying the matrix form of the Hamiltonian H by the vector Y. The product is assigned to the variable YDOT. We can describe these calculation in detail as follows:

**int** myfex_fnc_f1(**long int** *NEQ,

       **double** *T,

       dblcmplx *Y,

       dblcmplx *YDOT,

       dblcmplx *RPAR,

       **long int** *IPAR)

{

 simpleComplex<**double**> o0, o1, out0, out1;

  o0.re = 0.0;  o0.im = 0.0; o1.re = 0.0;  o1.im = 0.0;

 out0.re = 0.0; out0.im = 0; out1.re = 0.0; out1.im = 0.0;

 o0 = Y[0] * H[0]; o1 = Y[1] * H[1];

 out0 = o0 + o1;

 o0.re = 0.0; o0.im = 0.0; o1.re = 0.0; o1.im = 0.0;

 o0 = Y[0] * H[2]; o1 = Y[1] * H[3];

 out1 = o0 + o1;

 YDOT[0] = out0; YDOT[1] = out1;

 **return** 0;

}

The approach presented above naturally allows further optimization of the code during the operation of multiplying the matrix by the vector.

### 4.2 Trilinear Hamiltonian

In this example, we first assume that the Hamiltonian H is also time-independent. The simulated process is a time evolution of an optical parametric amplifier given by the following trilinear Hamiltonian [30]:

$$\begin{array}{c} H_{\text{SYS}}=iK\left(ab^{†}c^{†}-a^{†}bc\right). \end{array}$$ (20)

The symbols *a*, *b* and *c* stand for the boson annihilation operators corresponding to the pump, signal and idler fields respectively. The variable *K* represents the value of the coupling constant. For the purpose of the tests, we assumed that *K* = 1, and **i** represents an imaginary unit. The initial state of analyzed system is:

$$\begin{array}{c} |\psi_{0}{\rangle =|\alpha \rangle }_{a}{\left|0\right\rangle }_{b}{\left|0\right\rangle }_{c}, \end{array}$$ (21)

it is a coherent state for the pump mode (*a*), and the vacuum states for the signal (*b*) and the idler (*c*) modes.

We also utilize three expectation operators defined as:

$$\begin{array}{c} a_{0}=a⊗I_{b}⊗I_{c},\\ a_{1}=I_{a}⊗b⊗I_{c},\\ a_{2}=I_{a}⊗I_{b}\times c,\\ n_{0}=a_{0}^{†}a_{0},\;n_{1}=a_{1}^{†}a_{1},\;n_{2}=a_{2}^{†}a_{2}, \end{array}$$ (22)

where *a*, *b*, *c* still represent the boson annihilation operators with the same or different dimensionality. *I*_*a*_, *I*_*b*_ and *I*_*c*_ are the identity operators for fields: pump_mode (a), vacuum (b) and idler (c). The collapse operators are denoted as:

$$\begin{array}{c} c_{0}=\sqrt{2\cdot \gamma_{0}}a_{0},\\ c_{1}=\sqrt{2\cdot \gamma_{1}}a_{1},\\ c_{2}=\sqrt{2\cdot \gamma_{2}}a_{2}, \end{array}$$ (23)

where *γ*_0_ = *γ*_1_ = 0.1, *γ*_2_ = 0.4.

**Fig 6:**
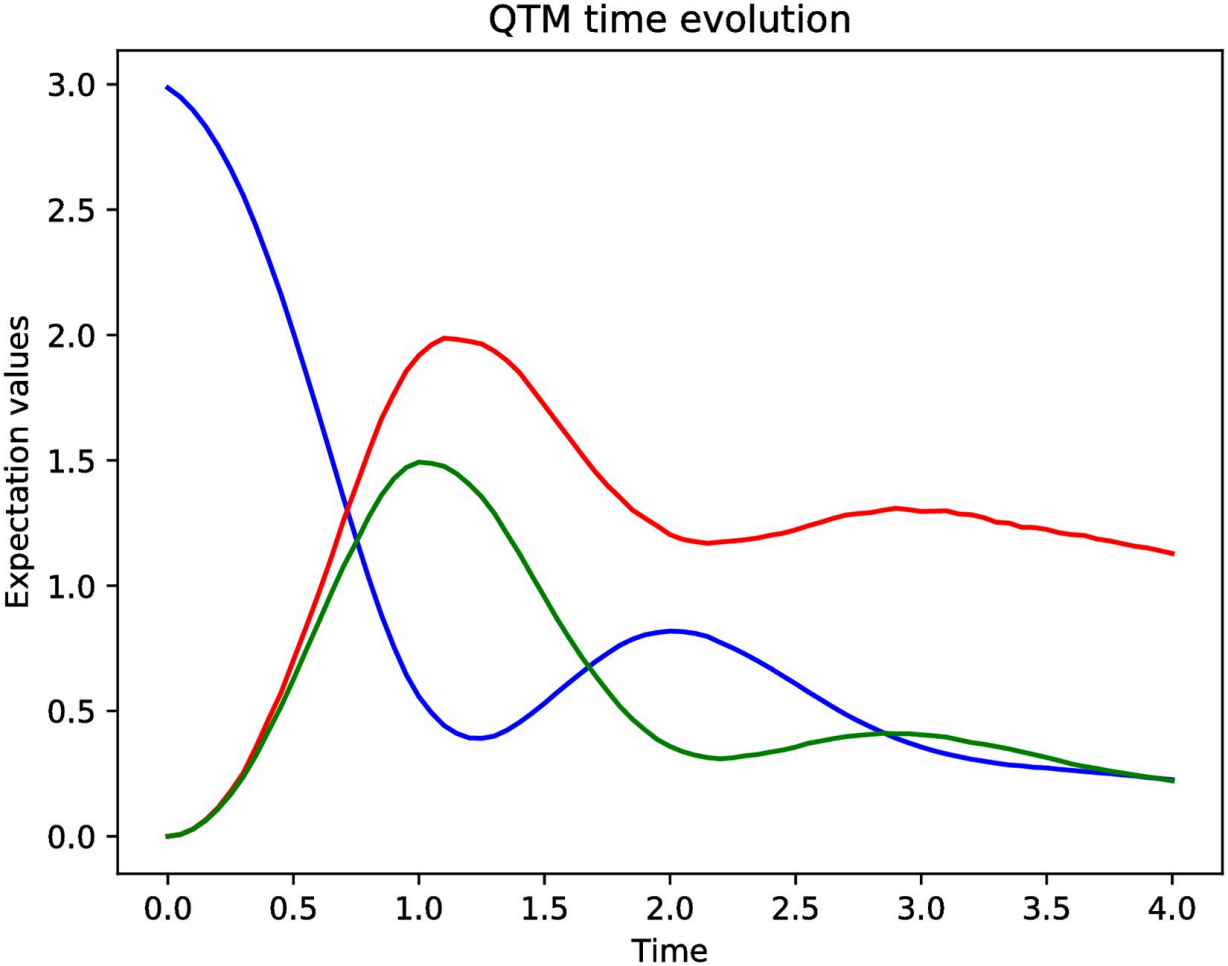
The expectation values describing the number of photons for different expectation operators (N0—Blue, N1—Red, N2—Green defined by Eq (22)) for a trilinear Hamiltonian—Eq (20).

The obtained expectation values of the photons’ number, during the experiment with 1000 trajectories, are presented in Fig 6.

**Fig 7:**
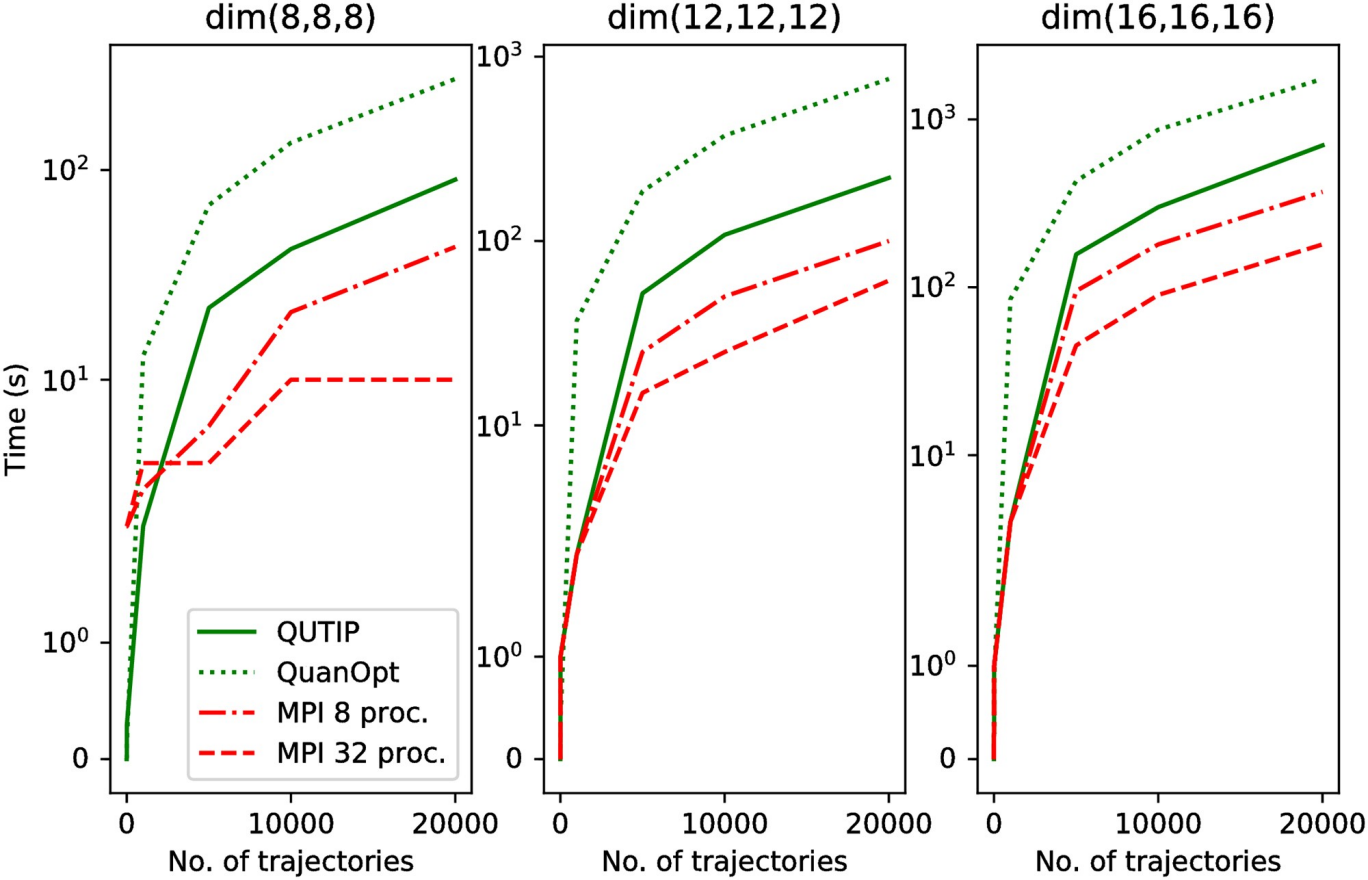
The duration of the simulation for the trilinear Hamiltonian. The experiments were run for the different number of trajectories with use of the QuTIP (four CPU cores were used), the QuantumOptics.jl (termed as QuanOpt, with only one CPU core used during the computations), and the QTM package (with two different numbers of the computational nodes).

Fig (7) depicts the simulations’ duration for the QuTIP, the QuantumOptics.jl and the QTM packages with the different number of trajectories. We also diversify the number of dimensions for the initial state in our experiments. The calculation for the MPI protocol was conducted with the use of nine computers equipped with Intel Core i7-4790K 4.0 GHZ processing units, and working under the Ubuntu 16.04 LTS operating system. Each processor has four cores, therefore, we were able to run the 32 MPI processes in eight computational nodes. The last processing unit serves as a master node.

We can observe a direct profit thanks to dividing tasks between many computational nodes. We should expect a significant speed-up of calculations if the number of computational nodes increases, therefore, we consider this to be a very good result. Especially for high-dimensional systems utilizing parallel computing and many computational nodes, the execution of the tasks directly translates into better performance i.e. shorter computation time with the QTM package. It should be emphasized that in the case of calculating 10.000—20.000 trajectories 32 MPI processes still offer sufficient computing power to increase the size of the problem or the number of trajectories. For the QuTIP package, this computation was run with the use of one computational node and the whole computing power was consumed. The same situation can be observed in the case of the QuantumOptics.jl package. It also utilizes the JIT system of compilation and offers a very efficient usage of a processing unit. However, only calculating many trajectories using the MPI technology causes significant reduction of the calculation time.

The simulation of a trilinear Hamiltonian requires using sparse matrices in order to maintain both low memory requirements and high performance. The QTM package offers basic data types to simplify the basic transformations and realize the calculation (also the definitions of operators may be given directly, as it was done for the Hamiltonian). A few selected steps connected with the preparation of the Hamiltonian representations are presented below:

// *Hamiltonian preparations*

**const int** N0 = 8, N1 = 8, N2 = 8;

**double** K = 1.0, gamma0 = 0.1, gamma1 = 0.1, gamma2 = 0.4;

**double** alpha_triham = sqrt(3);

uMatrix< simpleComplex<**double**> > d1(N0);

…

uMatrix< simpleComplex<**double**> > a0(N0*N1*N2),

        C0(N0*N1*N2), num0(N0*N1*N2);

…

uMatrix< simpleComplex<**double**> > H(N0*N1*N2);

…

destroy_operator(d1);

eye_of_matrix(d2);

eye_of_matrix(d3);

a0 = tensor(d1, d2, d3);

…

H = unity*K*(a0*dagger(a1)*dagger(a2)–dagger(a0)*a1*a2);

Heff = prepare_effective_H(H, C0, C1, C2);

H = convertToCSRMatrix(Heff);

…

vacuum = tensor(basis(N0,0), basis(N1, 0), basis(N2,0));

D = exp_of_matrix(alpha_triham * dagger(a0)

         − cojugate(alpha_triham)*a0);

alpha = D*vacuum;

…

r = mpi_main<100, Ntrj, WV_LD, WV_LD_SQR, 3>(argc, argv, 1, 0, 4, 1, 1, opt);

The changes also apply to the function calculating the right side of the ODEs. Luckily, only the function realizing multiplication has to be changed to the one supporting the CSR matrices. Therefore, the function calculating the right side of the ODEs is:

**int** myfex_fnc_f1(**long int** *NEQ,

       **double** *T,

       dblcmplx *Y,

       dblcmplx *YDOT,

       dblcmplx *RPAR,

       **long int** *IPAR)

{

  size_t i, j;

  **for** (i = 0; i < WAVEVECTOR_LEAD_DIM; i++)

  {

   YDOT[i].re = 0.0;

   YDOT[i].im = 0.0;

   **for** (j = H.row_ptr [i]; j < H.row_ptr [i+1]; j++)

   {

    YDOT[i] = YDOT[i] + (H.values[j] * Y[H.col_ind[j]]);

   }

  }

   **return** 0;

}

Referring to the function calculating the right side of the ODEs, it should be emphasized that the access to the current time value (parameter *T*) is possible, what facilitates using a time-dependent Hamiltonian.

The QTM package is implemented in the C++ language and the presented examples have to be compiled, therefore, the computation is carried out with a high efficiency.

### 4.3 Jaynes-Cummings model

The third example refers to the Jaynes-Cummings Model (JCM). This problem may also be simulated in the QTM package. In this case, a single trajectory corresponds to a computation run with the use of the master equation. Let us assume that the system’s dimension is *N* = 40. The dimensions of the operators are given in subscripts.

$$\begin{array}{c} g=1,\;\Delta =-0.1,\;\alpha =4.0,\\ a=d_{N}^{-}⊗I_{2},\;b=I_{N}⊗\sigma^{-},\\ H_{\text{SYS}}=\Delta a^{†}a+g\left(a^{†}b+ab^{†}\right), \end{array}$$ (24)

where *I*_2_ stands for the identity operator sized 2 × 2, *σ*^−^ represents the annihilation operator for Pauli spins. The values Δ and *g* represent coupling strength between an atom and a cavity. The initial state is expressed as:

$$\begin{array}{c} |\psi_{0}\rangle =|\chi_{N}^{\alpha }\rangle ⊗|1\rangle , \end{array}$$ (25)

where we make tensor product between a coherent state $\chi_{N}^{\alpha }$ and a single qubit in the state |1〉.

**Fig 8:**
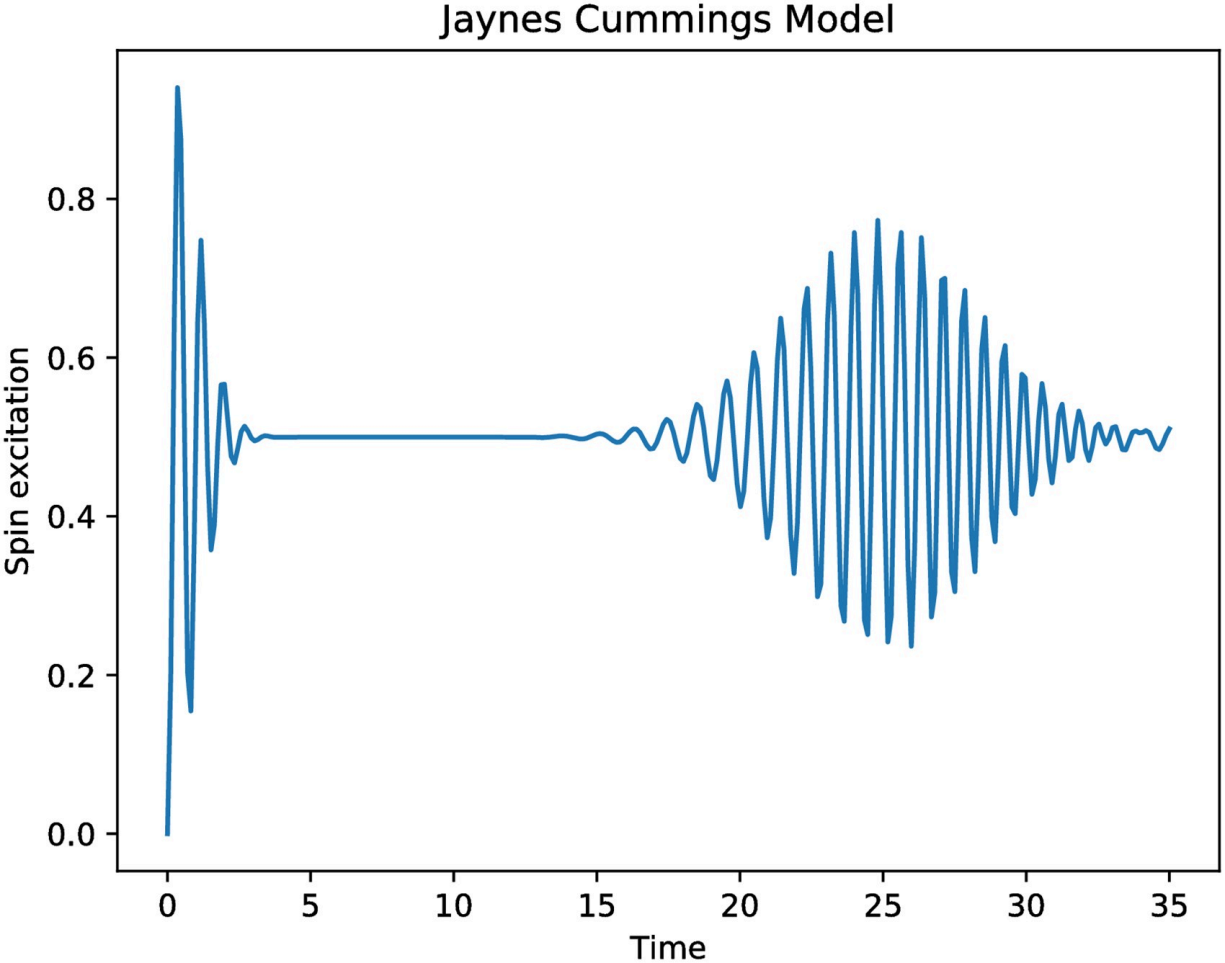
The values of the spin excitation in the JCM obtained after calculating one trajectory without using collapse operators.

The results obtained during the simulation of the JCM are presented in Fig 8. It should be emphasized that we utilize only one trajectory, and needed operators were represented by sparse matrices.

The most important pieces of the code for the JCM are listed as follows:

**const int** N = 40;

**double** g = 1.0, delta = -0.1, alpha = 4.0

uMatrix< simleComplex<**double**> > d1(N);

uMatrix< simleComplex<**double**> > eye2(2), sigmam(2);

…

sigmam_operator sigmam);

destroy_operator(d1); eye_of_matrix(eye2);

…

a = tensor(d1, eye2);

sigmaminus = tensor(eyeN, sigmam);

expect = dagger(sigmamnus)*sigmaminus;

H = delta * dagger(a) * a + g*(dagger(a)*sigmaminus + a * dagger(sigmaminus));

Heff = prepare_effective_H(H);

H = convertToCSRMatrix(Heff);

…

r = mpi_main<600, 1, N, N*N, 0>(argc, argv, 1, 0, 35, 0, 1, opt);

### 4.4 The birth and death of a photon in a cavity

The fourth and the last example refers to the convergency of the simulation of photon’s birth and death in a cavity, and it is based on paper [29]. Let *N* be a number of task’s dimensions, and *N* = 5. Then, the Hamiltonian and the collapse operators are expressed as:

$$\begin{array}{c} a=d^{-},\;H_{\text{SYS}}=a^{†}\cdot a,\;\left|\psi_{0}\right\rangle ={\left[0,1,0,0,0\right]}^{†},\;\kappa =1.0/0.129,\;t=0.063,\\ c_{0}=\sqrt{\kappa \cdot (1+t)}\cdot a,\;c_{1}=\sqrt{\kappa \cdot t}\cdot a^{†}, \end{array}$$ (26)

where *d*^−^ denotes the destroy operator, H—the Hamiltonian and *c*_0_, *c*_1_ represent the collapse operators, t—the temperature of the environment.

**Fig 9:**
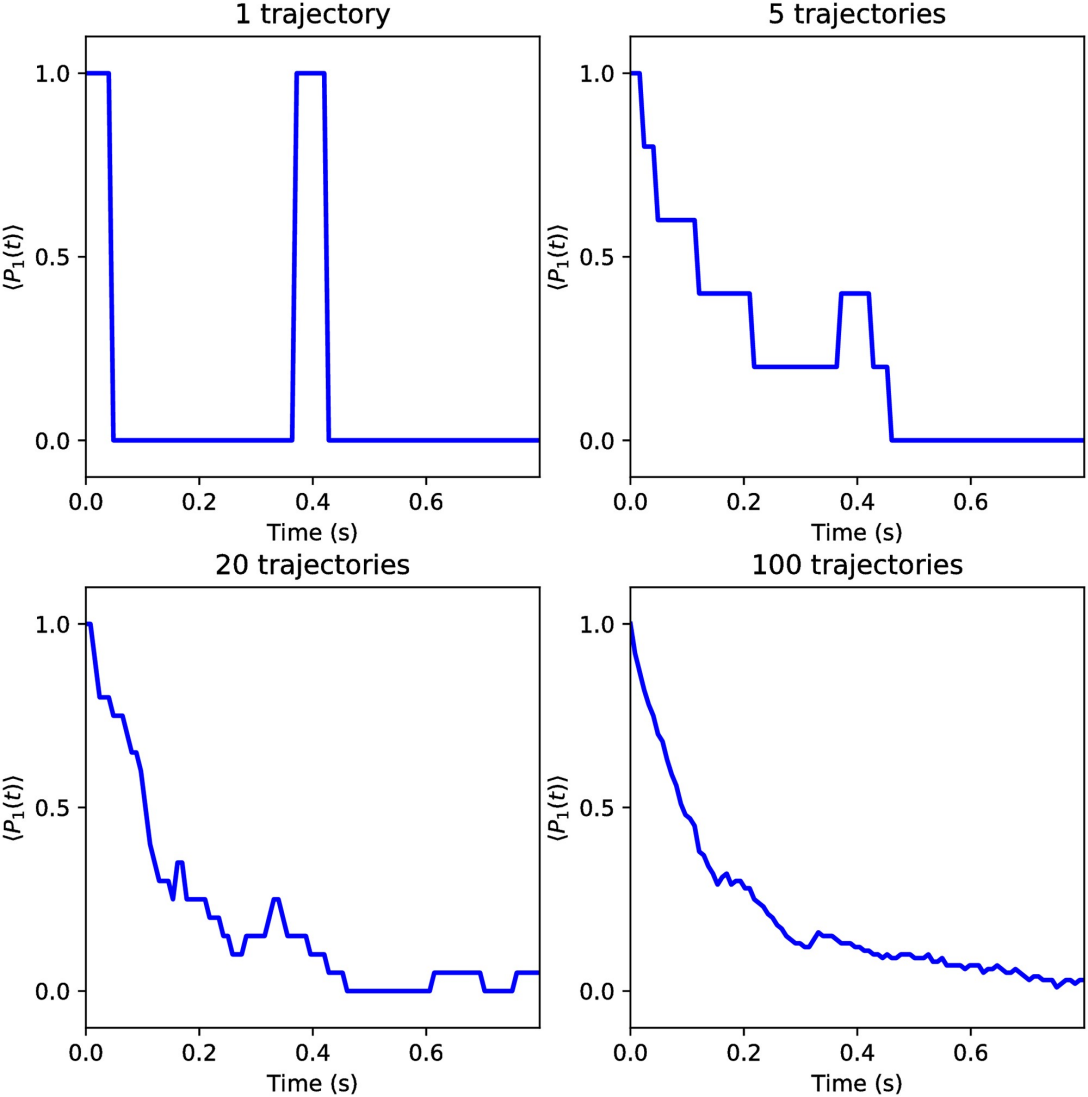
The probability of the decay for the one-photon state obtained with the different numbers of trajectories. Increasing the number of trajectories improves the accuracy of calculation.

Fig 9 shows that the number of generated trajectories improves the accuracy for a solved problem. Naturally, it also confirms the correctness of the realized QTM implementation.

The source code for this example is very similar to the previously presented pieces of the code. We utilize dense matrices because the system’s dimension is low, and it does not influence the performance.

## 5 Conclusions

A package which implements the Quantum Trajectories Method approach was presented in this article. The package is dedicated to examining the properties of the quantum open systems. The implementation is based on the MPI standard. The package was prepared in the C++ programming language, but the implementation does not require from the final user any advanced programming techniques. Utilizing the MPI standard allows using the package within systems realizing high-performance computing, but also small systems like personal computers because the communication processes introduced by the MPI package do not virtually increase demand of computing powers.

The current version of our package is the first version that has been made public. Naturally, further works and development are planned. We would like to implement a version based on the CUDA/OpenCL technology, which will be able to utilize computing powers of graphics processing units.

Recently, a new approach to the QTM was presented [2]. In next versions of the package, this novelty will be considered: the version of used QTM will be matched in accordance with a given problem in order to obtain a shorter time of calculations.
